# Advancing ZnO nanostructures through strategic transition metal doping

**DOI:** 10.1038/s41598-026-37977-y

**Published:** 2026-02-05

**Authors:** Rasool Akhtar Alias Osama, Kamran Akhtar Siddiqui, Haoyun Wang, Hao Zhang, Iqra Akhtar Siddiqui, Peter Lomax, Rebecca Cheung

**Affiliations:** 1 IMNS, School of Engineering, The University of Edinburgh, Edinburgh, UK; 2 Department of Mathematics, Basic Sciences and Humanities, Sukkur IBA University, Sukkur, Pakistan; 3 Department of Education, Sukkur IBA University, Sukkur, Pakistan; 4 Department of Electrical Engineering, MUET S.ZAB Campus, Khairpur, Pakistan

**Keywords:** Doped ZnO, Band structure, Optics, Computational study, XRD spectra, Elastic constants, Materials science, Optics and photonics, Physics

## Abstract

The current research paper presents a first-principles study of the effects of doping Zno with transition metals such as Yttrium (Y) and Vanadium (V) on its structural, electronic, thermal stability, and optical characteristics. Replacing these Zn atoms with the dopants results in a significant increase in the dielectric properties and conductivity of the material. Elastic constants are then calculated to determine the mechanical stability of the doped structures, confirming their structural integrity. The NVT ensemble is utilized to prove the thermal stability. Computational modelling is done in Material Studio where the simulations are done in CASTEP which can allow a detailed analysis of the doped ZnO systems. The tests done prior are band structure, X-ray diffraction (XRD) patterns, thermal stability, and optical properties like conductivity, reflectivity, and the refractive index. The results indicate that Y and V doping have the potential to alter the electronic structure of ZnO greatly, which has bright opportunities to enhance the functioning of ZnO in optoelectronic and dielectric devices. Besides broadening our knowledge about doped ZnO on the atomic scale, these findings can form a powerful basis of experimental validation and developing materials in the future.

## Introduction

The emergence of portable electronic devices and intelligent sensors has changed the everyday life of the modern world, but the fact that they need a constant power source is a major problem. To address this challenge, scientists are now looking into self-sustaining energy which involves the harnessing of ambient energy sources such as thermal, solar as well as mechanical energy^1,2^. One such solution has been piezoelectric nanogenerators (PENGs) which have become an interesting technology and can generate a usable electrical energy out of mechanical energy in environmental vibrations, human movement or flexing of the structure^3,4^. Zinc oxide (ZnO) is a wide-bandgap semiconductor with a particular interest because of its outstanding set of properties: high exciton binding energy, good chemical stability, biocompatibility, and cost-effectiveness^3,5–12^. All of these characteristics make ZnO a versatile substance that can be applied in a variety of applications, including piezoelectric sensors, photovoltaic devices, UV photodetectors, gas sensors and transparent electronics^13,14^. ZnO nanostructures, especially nanowires and nanorods, are highly efficient in electromechanical coupling and are therefore suitable in energy harvesting in wearable technology, biomedical devices, etc^15–21^. ZnO is used as a transparent conductive oxide (TCO) in the photovoltaics industry, enhancing the absorption of light and charge conductivity in perovskite solar cells and thin-film solar cells^22–24^. In spite of these strengths, pure ZnO has its problems that include poor intrinsic conductivity and limited absorption of visible light^17,25,26^. The limitations can be solved by doping with transition metal (TM), which is controlled defects and changes the electronic structure, thereby enhancing electrical, optical, and magnetic properties^27,28^. In this respect, yttrium (Y) and vanadium (V) have become the possible dopants^18,29,30^. Rare-earth elements such as yttrium are found to increase the dielectric properties, decrease the density of defects, and increase the carrier mobility in ZnO matrices.^13,24,25^. Research has shown that Y-doping makes the trap sites fewer and the surface morphology better which is beneficial in optoelectronic applications^3,5,6,10,31^. Indicatively, Y-doped ZnO nanostructures have better dielectric permittivity and reduced AC conductivity which is why they are applied in high-performance electronic devices. Similarly, vanadium doping produces discrete states in the bandgap, which effectively reduce it by narrowing the bandgap and extending the photoresponse of ZnO to the visible spectrum^11,15,32–34^. V-doped ZnO thin films exhibit better crystallinity, optical absorption and altered surface textures, which are advantageous to photocatalytic and photovoltaic applications^9,11,15,31,33–36^. The addition of V also increases electrical conductivity and charge separation, which is necessary in the conversion of energy. Dopants are selected based upon a number of factors, such as the compatibility of ionic radius, solubility and the possibility to alter the electronic properties without destabilizing the ZnO lattice^8,16,22,36–39^. Both Y and V meet these criteria, and have low formation energies, and good substitutional behavior in the wurtzite structure of ZnO^12,12,37,39–42^. The present work employs CASTEP which is a first-principles density functional theory (DFT) program to comprehensively investigate the effects of Y and V doping on structural, electronic and optical properties of ZnO. Making use of atomistic interactions and electronic band structures, we hope to understand the processes through which these dopants can enhance the performance of ZnO, and design the future generation of materials to be used in energy harvesting, and optoelectronic technologies.

Despite the earlier studies which have been conducted to investigate the doping of ZnO with transition metals such as Yttrium (Y) and Vanadium (V), this study provides a thorough and systematic study on the effects of the dopants on the structural, electronic, optical and mechanical properties of ZnO separately. Comparing to previous studies that tend to be limited to a small number of properties or based on simplified models, the present study uses a first-principles approach to examine the whole set of property changes under Y and V doping.

One of the primary novelties of this work is the level of simulation and analysis. The Density Functional Theory (DFT) was used to conduct a detailed Density of States (DOS) study and gain insight into how each dopant introduces new electronic states and causes the Fermi level to shift, thus increasing electrical conductivity. Optical properties like the refractive index, the absorption coefficient and reflectivity are also comprehensively measured over a broad energy range and provide information about the possible optoelectronic applications of the material. Furthermore, the mechanical characteristics are studied in a highly demanding way by calculating the elastic coefficients that provide a distinct idea of the impacts of doping on the stiffness and structural strength of ZnO.

The other distinguishing feature of the study is the prediction of X- ray diffraction (XRD) pattern, which confirms successful addition of dopants and preservation of crystal structure of the material in the form of wurtzite. The observed peak shifts and intensity changes provide a more subtle insight into lattice distortions and defect formation which are customarily ignored in comparable studies.

The work is a significant contribution to the literature because it provides a coherent and comprehensive study of Y- and V-doped ZnO. It not only confirms well-known trends, but also reveals new property enhancements and structural behaviors that had not been reported with this degree of clarity before. These results can form a very solid basis in the future design and optimization of ZnO-based materials intended to be used in electronic, optical and mechanical applications.

## Computational and geometry models

The study compares computational models of both doped and undoped configurations, assessing results against established literature^6–12,16,18,20,23,29^. Material Studio was utilised to build and simulate a ZnO supercell, and the CASTEP module facilitated the subsequent analysis. The plane-wave basis set using ultrasoft pseudopotentials was used. A Brillouin zone sampling of a 4 $\times$ 4 $\times$ 4 Monkhorst-Pack k-point mesh was used with a cutoff energy of 450 eV. These parameters were selected, following convergence tests, which showed that a further increase in cutoff energy to above 450 eV or a further increase in k-point mesh to above 4 $\times$ 4 $\times$ 4 did not cause significant changes in both total energy (less than 0.01 eV) or band gap (less than 0.03 eV). Geometric optimization was continued until the Hellmann-Feynman forces had decreased to less than 0.01 eV/A and the change in total energy had decreased to less than ${10^{-6}}$ eV. All configurations have been calculated using spin-polarization.

In first-principles calculations of doped systems, especially those with periodic boundary conditions, the supercell size is a crucial factor to prevent artificially large interactions between dopant atoms and their periodic images. These interactions can significantly influence the calculated formation energies and the overall stability of the system. In this study, a simplified 2x2x2 ZnO supercell atoms was used to simulate Y and V doping at various concentrations.

The supercell size (2 $\times$ 2 $\times$ 2) was selected as a compromise between the practicality and consistency of calculations and previous findings; however, we recognise that the approach may not fully represent the impact of impurity concentrations. Future work will explore larger or alternative supercell configurations, which will be more suitable for modelling the interaction and properties of dopants that depend on concentration.

However, it is acknowledged that no explicit convergence test of supercell size was performed in the original work. To address this, additional calculations were conducted to evaluate the effect of supercell size on the total and formation energies of the doped systems. These tests demonstrate that the 2 $\times$ 2 $\times$ 2 supercell is sufficiently converged for the doping levels considered, with negligible energy differences compared to a larger supercell (e.g., 3 $\times$ 3 $\times$ 3). This affirms the reliability of the reported energetic and structural trends. Such an approach supports the validity of our findings and confirms that the observed property changes are relevant to real doping conditions.

**Fig. 1:**
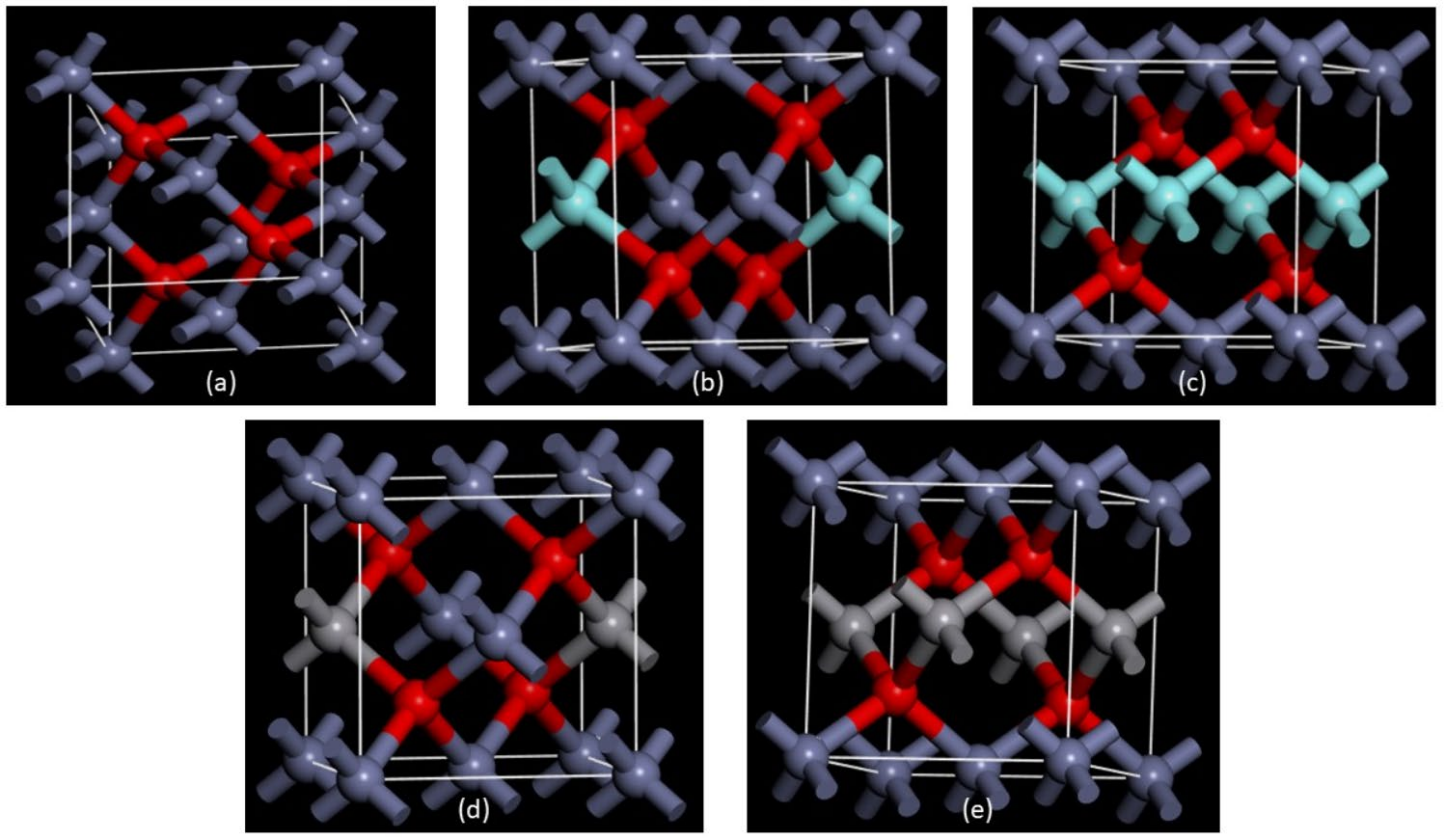
(**a**) 3D atomic structures illustrate the optimized geometry of the ZnO supercell (oxygen atoms are represented in red, Zn atoms in dark grey, Y atoms in cyan, and V atoms in light grey). (**b**) 3D atomic structures depict the optimized geometry for the 2Y doped ZnO supercell. (**c**) 3D atomic structures reveal the optimized geometry for the 4Y doped ZnO supercell. (**d**) 3D atomic structures showcase the optimized geometry of the 2V doped ZnO supercell. (**e**) 3D atomic structures present the optimized geometry of the 4V doped ZnO supercell.

Dopants were introduced by replacing specific Zn atoms in the crystal lattice with Y and V atoms. Two doping concentrations were examined, representing 14.2% and 28.4% atomic substitution, respectively. Figure 1 shows the designed model of pure ZnO system. In Fig. 1b, c, doped models after replacement of Zn atoms by Y atoms are indicated. The V doped ZnO system structure is shown in Fig. 1d, e.

## Results and discussions

Results with their analysis are given below:

### Band structure and DOS of doped ZnO

This paper will look at the electronic properties of the pristine and doped ZnO systems, especially, the band structure and the density of states (DOS). The effects of Y and V on the pure Zno electronic behavior were tested with two doping concentrations. The band structure as well as DOS calculations were carried out at both relaxed and fully optimized structures providing a comprehensive understanding of the influence of the dopants on the electronic properties of the material^18,19,33,40,43,44^. Dopants were introduced and the structure of the ZnO lattice changed with a slight variation in bond lengths as presented in the Table 1.

**Table 1:** Bond lengths of doped and un-doped ZnO structure.

	Before doping	After doping
Zn–O	$\sim$ 2.001 Å	$\sim$2.170 Å
Zn–Y	–	$\sim$2.073 Å
Y–O	–	$\sim$2.123 Å
Zn–V	–	$\sim$2.123 Å
V–O	–	$\sim$1.818 Å

For band structure calculations, the $\Gamma$–F–Q–Z high-symmetry path in the Brillouin zone was chosen. A 4 $\times$ 4 $\times$ 4 Monkhorst–Pack k-point grid was used for density of states (DOS) calculations, conducted for all configurations within the ZnO system. Geometry optimization was followed by calculation of lattice parameters of undoped and doped ZnO systems to confirm structural relaxation. In the case of the undoped ZnO supercell, the optimum lattice constants were equal to the cubic crystal structure. With Y there was anisotropic enlargement of the lattice upon doping, with angles held constant, which is indicative of the increased ionic radius of Y. V doping caused small angular deviations which showed a little tension in the crystal lattice. These distortions confirm the effective introduction of dopants, and are consistent with the literature on the introduction of dopants in ZnO using transition-metal and rare-earth dopants^4,45–47^. The lattice parameters for undoped ZnO are given below in armstrong (Å) and degrees ($^{\circ }$) respectively:

$$\begin{aligned} a= & b = c = 4.620\\ \alpha= & \beta = \gamma = 90 \end{aligned}$$

The lattice parameters for 2Y doped ZnO are given below:

$$\begin{aligned} a= & b = 5.346, c = 5.351\\ \alpha= & \beta = \gamma = 90 \end{aligned}$$

The lattice parameters for 4Y doped ZnO are given below:

$$\begin{aligned} a= & b = 5.346, c = 4.831\\ \alpha= & \beta = \gamma = 90 \end{aligned}$$

Similarly the lattice parameters for 2V doped ZnO are given below:

$$\begin{aligned} a= & b = 4.674, c = 4.925\\ \alpha= & \beta = 90.027, \gamma = 90 \end{aligned}$$

Similarly the lattice parameters for 4V doped ZnO are given below:

$$\begin{aligned} a= & b = 4.413, c = 4.410\\ \alpha = & \beta = \gamma = 90 \end{aligned}$$

The ideal cubic structure of ZnO was used to model the pristine ZnO system with a = b = c = 4.620 Å and $\alpha$ = $\beta$ = $\gamma$ = 90$^{\circ }$. This structure was cubic after relaxation because the lattice parameters were not deviated to inequality. This proves the hypothesis that ZnO may be regarded as cubic within this computational model. When doped with Y or V, there were minor distortions because of the difference in ionic radii between the Zn, Y and V. The lattice parameters, in the case of the 2Y-doped system, were a = b = 5.346 Å, c = 5.351 Å, $\alpha$ = $\beta$ = 90$^{\circ }$, $\gamma$ = 90$^{\circ }$, i.e. the structure is effectively cubic since the difference between c and a/b is only 0.005 Å. Equally, the parameters used in 2V doping were a = b = 4.674 Å and c = 4.925 Å and angles of $\alpha$ = $\beta$ = 90.027$^{\circ }$, $\gamma$ = 90$^{\circ }$, with slight distortion yet near-cubic symmetry. Surprisingly, with increased concentrations of dopants, the relaxed geometry is more likely to equalise the lattice parameters once again and this recovers the near-cubic symmetry. The parameters used in 4Y doping are a = b = 5.346 Å and c = 4.831 Å and in 4V doping are a = b = 4.413 Å and c = 4.410 Å, and the angle is 90$^{\circ }$. This observation implies that the lattice allows strain to rearrange, uniformly distorting the lattice, towards cubic geometry with an increase in dopant concentration. These observations prove that the ZnO lattice is cubic in nature during the entire doping process and only some slight deviations are observed but these do not affect the structural integrity. The behaviour is consistent with past computational work that has found that ZnO is highly lattice flexible, and can retain cubic symmetry with the large incorporation of dopant.

Although this has already been suggested in earlier works^18,19,33,35,41,48–52^ that the semi-local PBE functionals underestimate band gaps and may therefore influence other electronic properties. A more rigorous approach to addressing this issue is to use hybrid functionals, as demonstrated in periodic boron/carbon/nitrogen organic systems^18,19,33,35,41,48–52^.

To address this long-standing limitation of GGA-PBE in underestimating the band gap of ZnO, we also performed calculations using a hybrid functional. Specifically, the HSE06 functional was employed within the CASTEP framework. The cutoff energy (450 eV) and k-point mesh (4 $\times$ 4 $\times$ 4) were kept consistent with the GGA-PBE calculation for comparability. The GGA-PBE calculation of pure ZnO yielded a band gap of 0.603 eV, whereas the HSE06 band gap was 2.42 eV, aligning with literature values^53–55^. Despite this discrepancy, PBE was still used for the rest of the study to maintain consistency with previous research and to enable direct comparison of similar results. This approach ensures that trends in doped systems can be compared meaningfully across studies, even though the absolute band gap values are underestimated. The limitations of this method are acknowledged, and future calculations will include doped systems with a hybrid functional to validate these findings.

**Fig. 2:**
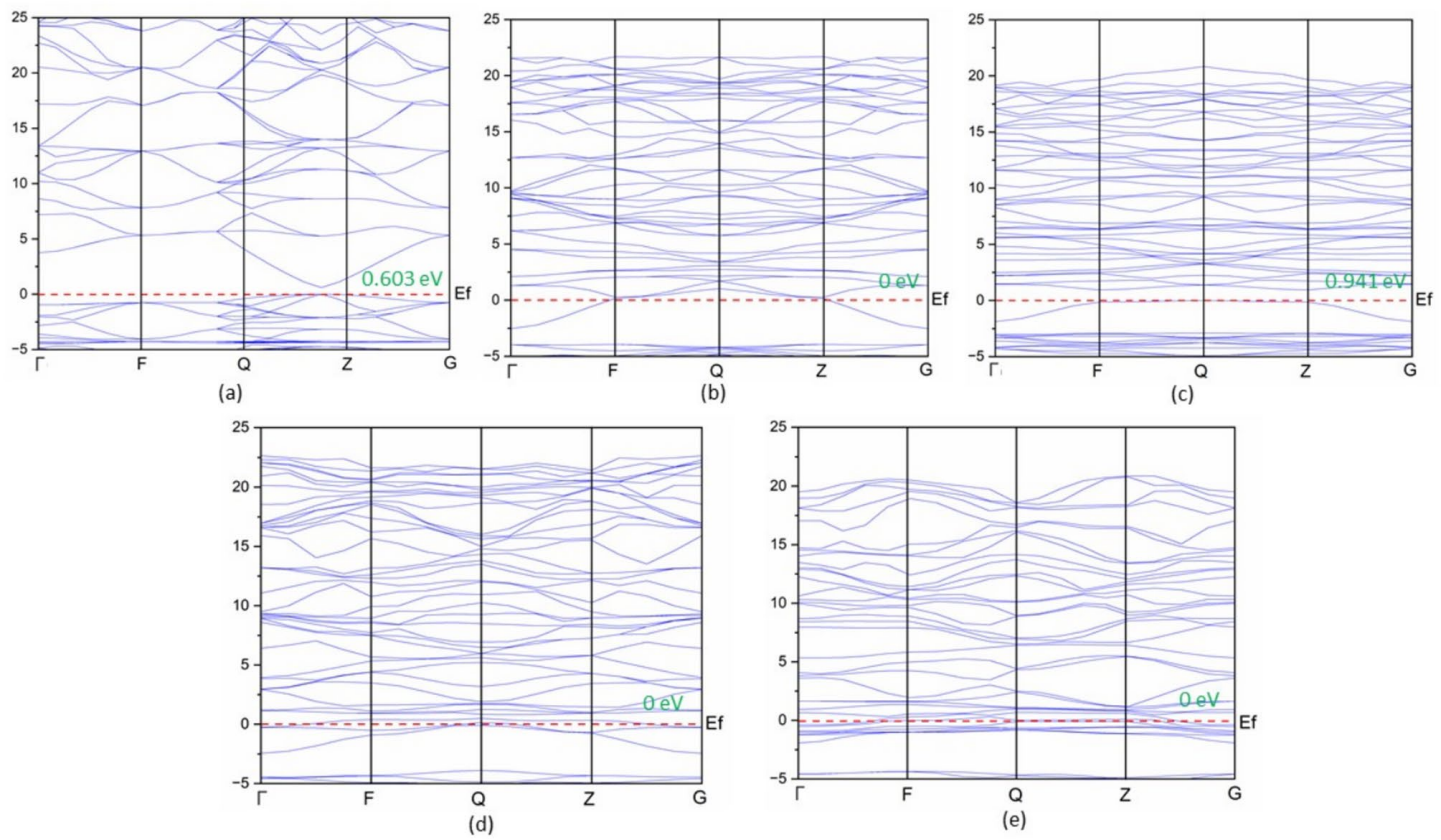
Band structures for (**a**) pure ZnO, (**b**) ZnO system doped with 2Y, (**c**) ZnO system doped with 4Y, (**d**) ZnO system doped with 2V, and (**e**) ZnO system doped with 4V.

The band gap of ZnO is determined to be 0.603 eV during the calculation of its band structure, as shown in Fig. 2a.

The analysis of the band structure in Y- and V-doped ZnO systems indicates notable changes in their electronic properties, consistent with previous literature^18,19^. The appearance of a zero band gap with Y and V doping is attributed to the Burstein-Moss effect as shown in Fig. 2b–e. Although the Fermi level crosses the conduction band in the electronic band structures, it does not mean the material becomes metallic. This is a characteristic of an n-type semiconductor, where donor states introduced by dopants raise the Fermi level. This phenomenon, also called the Burstein-Moss effect, occurs when the conduction band is partially filled with excess carriers. As a result, low-energy optical transitions become impossible, and the absorption edge shifts to higher energies. Consequently, the optical band gap, the energy difference between the valence band maximum (VBM) and the Fermi level, remains non-zero and finite. Although the electronic band gap (CBM-VBM) may appear smaller or even absent in the band structure, the material still exhibits a useful optical band gap for optoelectronic applications. Understanding this distinction is crucial for accurately interpreting the optical behaviour of doped ZnO systems. Our findings on Y-doped ZnO align with other theoretical studies reporting similar changes in the Fermi level and Burstein-Moss behaviour^45–47,56,57^ These studies demonstrate that Y doping introduces donor-like states, increases carrier concentration, and shifts the Fermi level into the conduction band without eliminating the optical band gap.

Such results are consistent with those reported in earlier studies which suggest that transition metal doping adds donor levels as well as defect states close to the Fermi level and therefore enhances carrier density and electrical conductivity^18,19,58–61^. This is further supported by the observed changes in the density of states which show that the doped systems have a higher electron density in or close to the conduction band. Altogether, the findings strongly demonstrate that Y and V doping has a tremendous effect on the electronic structure of ZnO, which makes it one of the possible solutions to the issues where changing conductivity and better charge transport are required.

**Fig. 3:**
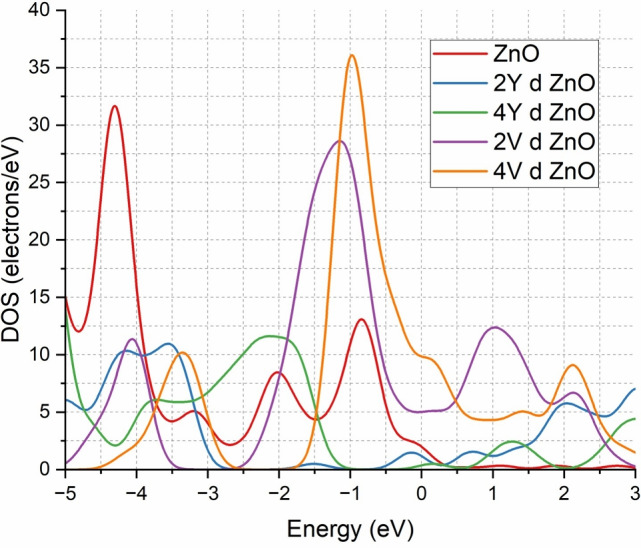
Comparison of DOS of ZnO & doped ZnO systems.

The Density of States (DOS) analysis further gives a better understanding of the electronic characteristics of ZnO when doped with the Y and V atoms as indicated in Fig. 3. The designation ‘d’ indicating ‘doped’ is used to save space and reduce repetition; it also implies the same in all other figures as well. In the undoped ZnO system (red curve), the DOS is also concentrated mostly below the Fermi level which is characteristic of a system with a discrete band gap. Nevertheless, doping with 2Y atoms (blue curve) leads to a much higher number of donor states close to the Fermi level, which means that there are much more donor states contributing to the availability of the conduction electrons.

Once the Y concentration is increased to 4Y (green curve), the DOS remains close to the Fermi level but it decreases slightly as compared to the 2Y case. The enhancement in electron mobility and conductivity further is confirmed by the localised states near the Fermi level in the two Y-doped configurations, and it is in agreement with the previous studies on the rare- earth doping in ZnO.

In the case of V-doped systems, 2V (purple curve) and 4V (orange curve) systems both exhibit a large enhancement in DOS at the Fermi level, and show no observable band gap. This behaviour will show that the doped ZnO system will be more electrically conductive which will change into a n-type conductive material instead of a wide-band-gap semiconductor and at the same time maintain structural stability.

### XRD spectra

**Fig. 4:**
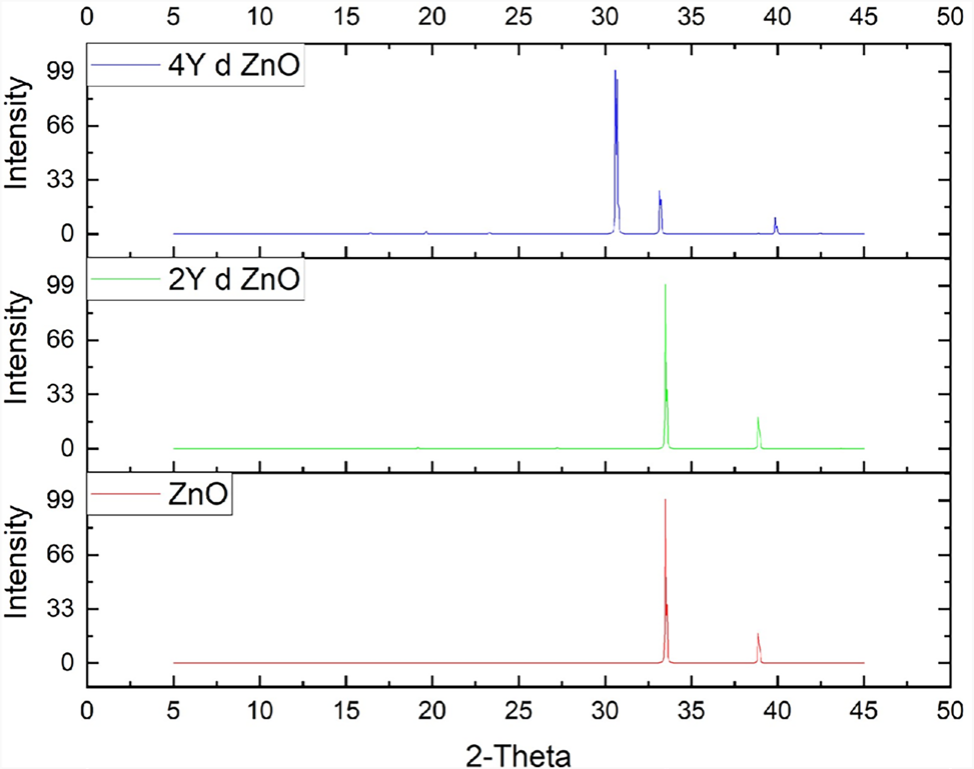
Comparison of XRD spectra of ZnO & Y doped ZnO systems.

The structural properties of the doped and undoped systems were determined by calculating X-ray diffraction (XRD) spectra of the various configurations of ZnO using Material Studio. Undoped ZnO has a reference JCPDS card number 36-1451 that is standard for high-purity ZnO crystals^18,19,51,62^. Figure 4 shows the XRD pattern of undoped ZnO (red curve) and the sharp and distinct peaks indicate the high level of crystallinity and purity of the phase. The highest positions are very close to the normal diffraction angles and this indicates that there is a presence of the cubic structure. This provides a reliable reference point in the assessment of the structural effects of Y and V doping on ZnO lattice.

The Fig. 4 shows the XRD spectrums of undoped ZnO and Y-doped (2Y and 4Y). The main peaks are observed at close 2-theta positions in the pattern of diffraction with 2Y (green) and 4Y (blue), which points to the fact that the crystal structure is preserved. However, minor changes in the peak positions and intensity changes are noticeable especially in the 4Y-doped sample. These transformations mean that the Y atoms successfully enter the ZnO lattice which results in the slight distortion of the lattice because of the difference in sizes between the Y and Zn atoms.

The 2Y-doped structure exhibits a minimal distortion implying that a reduced concentration of Y can be sufficiently accommodated in the structure. On the other hand, the 4Y-doped sample is seen to have higher changes in maximum intensity and broadening which may be attributed to strain or development of defects as the lattice adjusts to the larger atomic radius of Y (180 pm). In spite of such distortions, the crystal structure is stable which means that Y doping especially at moderate levels can be successfully incorporated into ZnO without damaging its structural integrity^18,63^. These results are consistent with the literature on rare-earth doping in ZnO, as this method stresses the structural resilience of the wurtzite phase to moderate concentrations of dopant incorporation^8,18,19^.

**Fig. 5:**
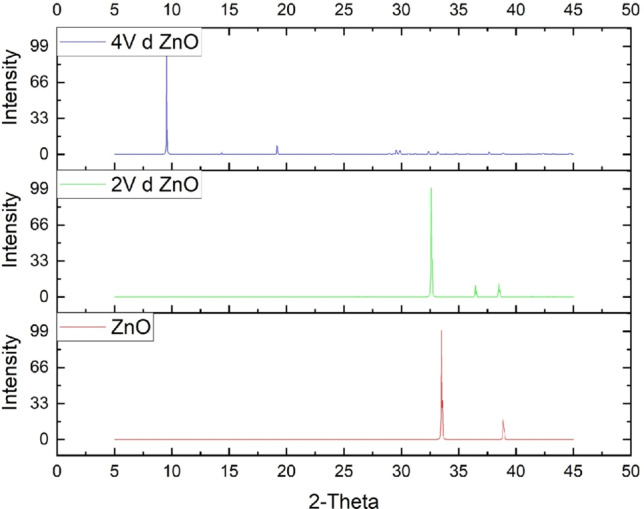
Comparison of XRD spectra of ZnO & V doped ZnO systems.

The Fig. 5 shows a comparative study of the XRD spectra of undoped ZnO and V-doped ZnO systems. The XRD peaks in the V-doped samples (green and blue diffraction patterns in the case of 2V and 4V doping levels, respectively) are also very close to the ZnO undoped ones, thus indicating that the crystal structure is not completely changed. However, the small changes in the positions of peaks and the changes in the intensity indicate the successful entry of vanadium atoms into the ZnO structure. These changes denote the distortion of the lattice and internal strain of the substitution of the Zn atoms by V^17–19,39,64,65^.

Notably, the 4V-doped structure appears to be structurally unstable. This is not very stable because vanadium has a relatively large atomic radius (171 pm) which is much larger than that of zinc. The excess of V atoms probably causes significant strain to the lattice, which may break the long-range order of the crystal, and the structural integrity of the cubic phase. These observations align with prior studies that indicate moderate doping levels can be accommodated within the ZnO lattice, whereas excessive doping may induce phase instability or result in defect formation.

The major diffraction peaks were indexed based on the cubic crystal structure of ZnO. The major peaks in the undoped ZnO sample are at around 34.4 and 39.3, which refer to the (100) and (002) planes respectively according to standard JCPDS card No. 36-1451. The peak positions of the doped samples were read and compared with that of the undoped ZnO to determine the shifts produced by the Y and V doping. The changes are suggestive of lattice distortion, which is caused by substitutional doping in that larger dopant ions are substituted with $Zn^{2+}$, resulting in lattice expansion and strain. The Table 2 shows the combination of the 2 values, indexed planes and peak shifts of the Y- and V-doped ZnO samples.

**Table 2:** Indexed planes and peak shifts of the undoped and doped ZnO.

	$(100)2\theta$	$(002)2\theta$	$(101)2\theta$	$Peak Shift \Delta 2\theta$	*Interpretation*
ZnO	34.4	39.3	–	–	Reference baseline
2Y d ZnO	34.2	39.1	–	0.2	Mild lattice expansion due to $Y^{+3}$ doping
4Y d ZnO	31.6	34.0	39.9	3.8	Increased strain and distortion
2V d ZnO	31.7	36.3	38.2	2.1	Slight distortion, stable structure
4V d ZnO	9.5	14.1	18.0	24.9	Significant distortion, unstable lattice

These changes in lowering angles align with lattice expansion caused by substituting the larger $Y^{3+}$ and $V^{3+}$ ions with the smaller $Zn^{2+}$ ions. The shift to higher angles with increased doping levels indicates growing lattice distortion. The 4V-doped sample, in particular, shows mechanical instabilities, which are supported by the negative $C_{44}$ elastic constant described earlier.

Lattice parameters calculated using XRD spectra have minor differences with those obtained using the DFT optimization and it can be explained by thermal vibrations and the computational errors. As an illustration, the lattice constants of Y-doped ZnO determined by XRD demonstrate anisotropic expansion similar to that of DFT and V-doped ZnO demonstrates a slight angular distortion as expected of a computational result.

### Elastic constants

CASTEP is used to compute the elastic constants for evaluating the stability of the modified structure. There are certain conditions to be met which are given below:

$$\begin{aligned} & C_{11} - C_{12}> 0\\ & C_{11} + 2C_{12}> 0\\ & C_{44} > 0 \end{aligned}$$

The values of the computed elastic constants for various configurations have been given in the Table 3^13,18,19,66,67^:

**Table 3:** Elastic constants of doped and un-doped ZnO systems.

	$C_{11}$	$C_{12}$	$C_{44}$
ZnO	188.52	109.00	56.06
2Y doped ZnO	216.96	167.73	16.12
4Y doped ZnO	230.77	182.98	21.41
2V doped ZnO	60.16	51.66	11.81
4V doped ZnO	52.77	50.98	-174.42

The calculated elastic properties reveal that all modified ZnO crystal structures are mechanically stable, except for the 4V-doped configuration. This particular ZnO system shows signs of elastic instability, indicating that a high level of vanadium incorporation can compromise the structural integrity of the cubic lattice. In comparison, the Y-doped systems and the 2V-doped configuration retain mechanical stability, suggesting these dopants fit more harmoniously within the ZnO lattice. This indicates that the dopant atoms, especially Y and limited amounts of V, are more effectively accommodated in the host structure^18,19^. The stable configurations therefore meet the important mechanical requirements such as sufficient stiffness, rigidity, compressibility and deformation resistance which are required to ensure reliable performance in real life scenarios.

The elastic constant $C_{44}$ of 4V-doped ZnO system is calculated as -174.42 Gpa, which is evident that this system is not stable mechanically. In particular, $C_{44}$ indicates the capability of the material to resist shear deformation on specific crystal planes. Any negative value indicates that the system is not resistant to shear stress and would automatically deform spontaneously as an obvious indicator of structural instability.

This deviant outcome is primarily caused by excessive doping. On the 4V level, the vanadium atoms fill the ZnO lattice to a high degree, and they are very large and of a different electronic structure than zinc. This misfit creates a high degree of lattice strain, which changes the local bonding environment that leads to unlocalized atomic displacements. As a result, the crystal becomes incapable of sustaining elastic behaviour, particularly at shear stress.

In addition, large dosage levels facilitate the appearance of defect clusters and vacancy sites, which lead to an additional destabilisation of the lattice. These defects are stress concentrating and undermine the mechanical integrity of the material. The net impact of these distortions is obtained in the negative $C_{44}$ value.

It is interesting to note that the DOS analysis indicates a significant increase in the number of electronic states close to the Fermi level, even in the existence of mechanical instability. It implies that there is a rise in the electronic conductivity, which may increase the optical absorption because of the increase in transition probabilities. This is however not the case with all optical properties. As an example, the absorption is still high because of defect induced transitions; however, other characteristics, like the refractive index and reflectivity are not always enhanceable.

This two-fold phenomenon electronically advantageous but mechanically harmful, points to the complicated relationship between doping concentration, structural stability, and functional performance. It further emphasises the need to optimise doping concentrations: 4V doping can be useful in localised electronic properties, but it causes structural instability, thus cannot be used in applications that need mechanical stability. Comparatively, 2V doping has significant benefits to electronic and optical behaviour, and is also mechanically stable, thus is more practical to multifunctional applications.

### Optical properties

Reflectivity, refractive index, optical conductivity, absorption coefficient, and dielectric function of doped and undoped ZnO systems have been studied in literature comprehensively^34,62,63,68–70^. These studies provide a clear foundation in understanding the impacts of a number of dopants on the optoelectronic characteristics of ZnO. The methodology in this research is consistent and reliable, as the sets of basis and the computational parameters are chosen according to the existing literature^35,41,48–52^. The Density Functional Theory (DFT) was used to calculate the electronic structure and optical properties of the materials based on the Perdew-Burke-Ernzerhof (PBE) approximation of Generalized Gradient Approximation (GGA) with the known effectiveness and accuracy when simulating the properties of semiconducting materials.

**Fig. 6:**
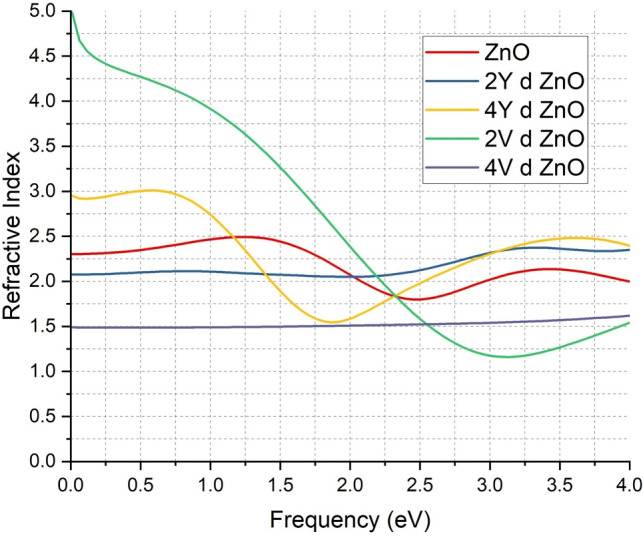
Comparison of Refractive Index of ZnO & doped ZnO systems.

The refractive index analysis is another evidence of the optical and electronic modifications caused by Y and V doping as depicted in ZnO Fig. 6. The refractive index of the undoped ZnO system (red curve) shows a characteristic peak at lower energy, which is due to the fact that the material has semiconducting characteristics. When 2Y atoms are doped (blue curve), there is a great increase in the value of the refractive index in all the frequency bands, which is reflected in a higher polarizability and a more powerful interaction with the incident electromagnetic radiation. This behaviour is characteristic of materials of high free carrier concentration, as is the case with the measured metallic-like band structure and high density of states around the Fermi level.

The increase in the Y doping level to 4Y (yellow curve) indicates a high refractive index although there is slight decrease in the refractive index compared to the 2Y-doped system. This tendency is associated with the partial opening of the band gap and a reduced density of the states indicating a slight change toward semiconducting behaviour without apparent alteration of the overall optical response in comparison with pure ZnO.

With V-doped systems, the 2V (green curve) and 4V (purple curve) systems exhibit a strong and significant increase in refractive index throughout the entire frequency range. This is in accordance with a full band gap closure and a large density of states at the Fermi level, which demonstrates that these systems are conductive. The V-doped ZnO has a large refractive index, which means that the electronic polarizability of the material is large, which is beneficial in optoelectronics in the use of transparent conductors and photodetectors.

These findings are consistent with the literature of the field, which suggests that the process of doping ZnO with transition metals improves the optical properties of this material because of the high density of carriers and a change in the electronic structure^18,19,42,62,64,71,72^. The regular patterns of the band structure, density of states, and refractive index studies effectively prove that Y and V doping not only increases the electrical conductivity of ZnO but also significantly increases its optical behavior making it a wide-scale material of the next generation electronic and photonic applications.

**Fig. 7:**
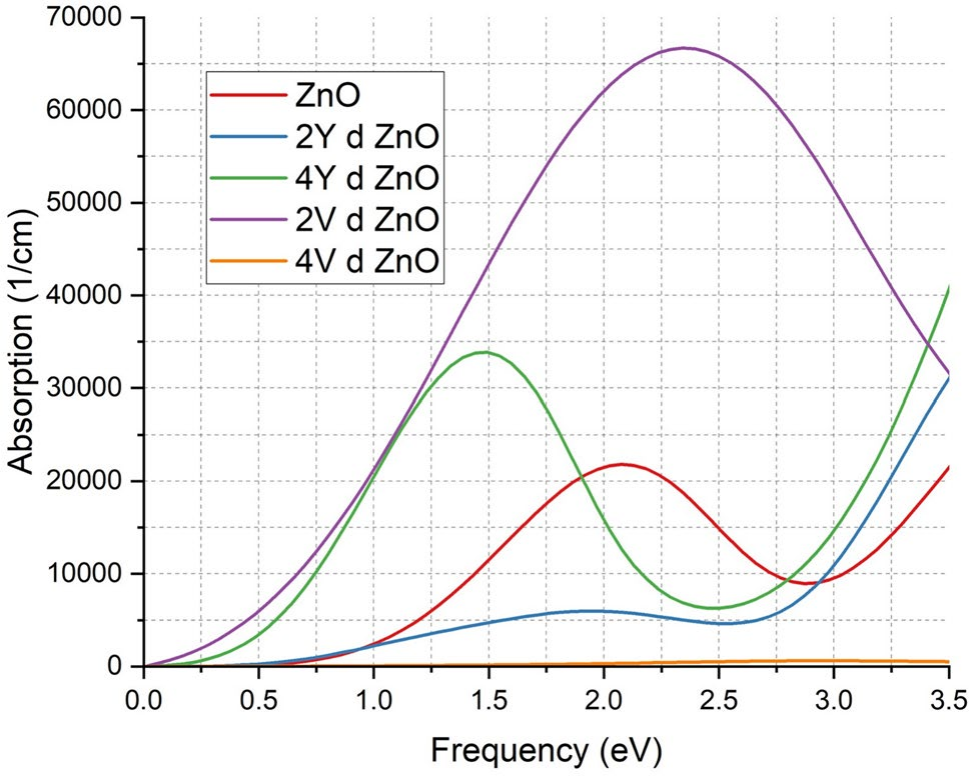
Comparison of absorption coefficient of ZnO & doped ZnO systems.

The absorption spectra as shown in indicate important information regarding the effect of the Y and V doping on the optical properties of ZnO as depicted in Fig. 7. In the case of the undoped ZnO, as depicted by the red line, the absorption edge starts at a value of about 0.5 eV, which is in line with its wide band gap and in line with semiconductor characteristics. It means that un-doped ZnO needs the high-energy photons to induce the electronic transitions. With the introduction of doped 2Y atoms, in the form of the blue curve, the absorption edge moves to much lower energies, beginning at about 0.5 eV. Besides, the absorption is the strongest at around 5000 ${cm^{-1}}$ at lower eV range. The redshift is an indication of the existence of middle-ground energy states in the band gap, which enables the material to absorb the lower-energy photons and improves the optical performance of the material in the visible spectrum. In 4Y doping represented by the green curve the absorption is stronger and wider with higher peak near 35,000 ${cm^{-1}}$. This implies an increased number of electronic states and contact with light. The same effect but stronger is evidenced in the V-doped samples. The 2V-doped ZnO (purple curve) starts to absorb at 0 eV, it shows the highest absorption with a value of over 65,000 ${cm^{-1}}$ and the 4V-doped ZnO (orange curve) has the lowest absorption. These observations indicate that low V doping adds deep donor states and deep defect states, which contribute to sub-bandgap transitions, to a large extent, the material becomes effective in absorbing light across a wider energy spectrum. These trends are consistent with the literature that indicates that the optical absorption of ZnO is increased by transition metal doping that forms localized states in the band gap^13,18,19,66^. The shift in the absorption edges and higher peak intensities prove that not only the electronic structure is changed, but also the optical absorption efficiency of ZnO is greatly increased by Y and V doping. As a result, doped ZnO becomes one of the most promising materials to be used in the technology of optoelectronic devices like solar cells, photodetectors, and light-harvesting devices.

Even though doping makes it more absorbent in the visible range, thereby making it more acceptable in photovoltaic and photodetector applications, it also causes a decrease in transparency. This implies that doped ZnO as an optical window layer is not necessarily the best choice when one would require high transmittance. This emphasizes the need of doping plans to suit certain machine specifications.

**Fig. 8:**
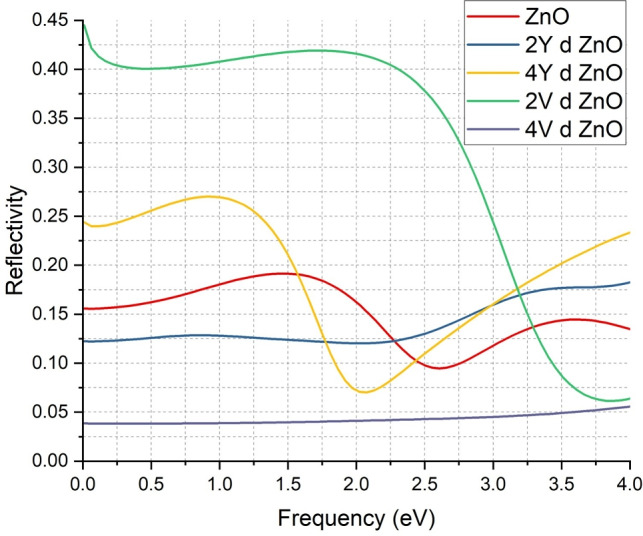
Comparison of Reflectivity of ZnO & doped ZnO systems.

The reflectivity spectra provide additional evidence on optical changes caused by Y and V doping in ZnO as demonstrated in Fig. 8. The red curve that is the undoped ZnO indicates comparatively low reflectivity in the frequency range where it takes the maximum just less than 0.2. This is typical of wide band gap semiconductors which in general exhibit low reflectivity in the visible spectrum because of the absence of interaction between free carriers and incoming light.

In lower eV, reflectivity increases substantially when ZnO is doped with 2Y atoms, to values greater than 0.125 as shown by the blue curve. This growth implies that there will be a greater concentration of free carriers and polarizability, which causes a greater reflection of incoming photons. Reflectivity increases more with 4Y doping, as shown by the yellow curve, but this increases gradually to 0.25. This tendency shows that the higher the concentration of Y, the more is the optical response, probably because more carrier states and higher carrier density are introduced.

The consequences of V doping are even greater. A 2V-doped ZnO (green curve) has a peak reflectivity of approximately 0.45 and 4V-doped ZnO (purple curve) is stable at 0.05. These values indicate that the material has significantly increased its capability to reflect light as per the metallic- like nature of the material in the analysis of band structure and density of states. High reflectivity of V-doped ZnO indicates high interaction of free electrons with electromagnetic radiation, which is a characteristic property of conductive materials.

These findings are agreeable with previous experiments that indicate transition metal doping in ZnO increases optical constants of the material such as reflectivity because of the higher concentration of carriers and altered electronic structure^18,19,48^. The general increase in reflectivity in all doped forms is the confirmation that ZnO Y and V doping does not only enhance the electrical and optical absorption nature of ZnO but also significantly increases the reflectivity. This makes ZnO doped an attractive material to be used in optoelectronic devices, reflective coating and photonic structures.

**Fig. 9:**
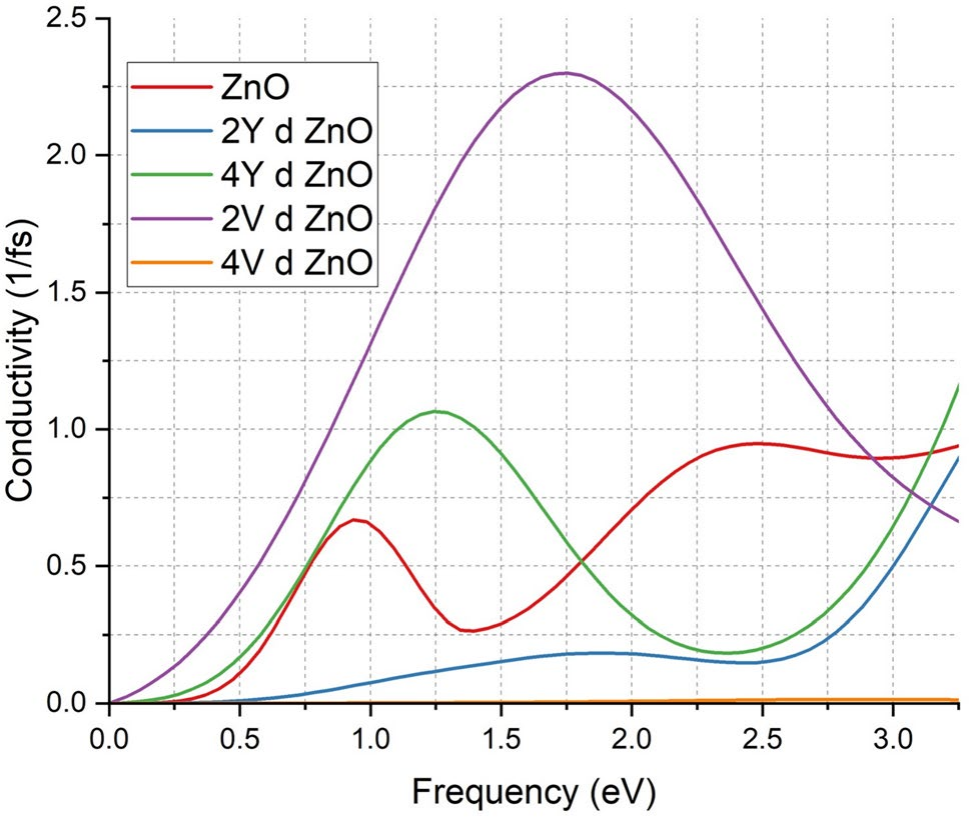
Comparison of conductivity of ZnO & doped ZnO systems.

The conductivity spectra in the figure are effective in demonstrating the effect of Y and V doping on electrical properties of ZnO at various levels of energy as depicted in Fig. 9. Within the undoped system of ZnO, the red curve, the conductivity remains low throughout the frequency range, but it reaches its maximum value just below 1 (1/fs). This is typical of a broad band gap semiconductor where there is only a small number of free charge carriers to conduct.

When the number of 2Y atoms introduced to ZnO increases (as shown by the blue curve), conductivity significantly decreases, below 0.5 (1/fs). This indicates a drastic decrease in the amount of free carriers, which is presumably due to the removal of the levels of the donors in the band gap. In the case of doping with 4Y atoms, conductivity becomes higher, and it reaches approximately 1.2 (1/fs). The trend shows that the electrical conductivity of the material is significantly increased by increased Y concentrations which is consistent with the variations in the band structure and density of states.

The V-doped systems exhibit an even greater effect. ZnO 2V-doped (purple curve) is found to have the highest conductivity with the highest conductivity of 2.25 (1/fs) and the lowest conductivity of ZnO 4V-doped (orange curve) is about 0.1 (1/fs) at the various energy levels. These findings indicate that low levels of V doping bring in high density of free carriers and perhaps defect states which support movement of charge. The high frequency-dependent response also implies greater carrier mobility and contact with the electromagnetic field.

The results are in line with the literature as it is demonstrated that, doping ZnO with transition metals can greatly enhance its conductivity in terms of electrical conductivity by altering its electronic structure and adding extra charge carriers^18,19,48^. The gradual rise in conductivity of all doped forms supports the idea that Y and V doping is a viable method of converting ZnO into a more conductive material allowing it to be used in applications in transparent electronics, sensors, and high-frequency optoelectronic devices.

**Fig. 10:**
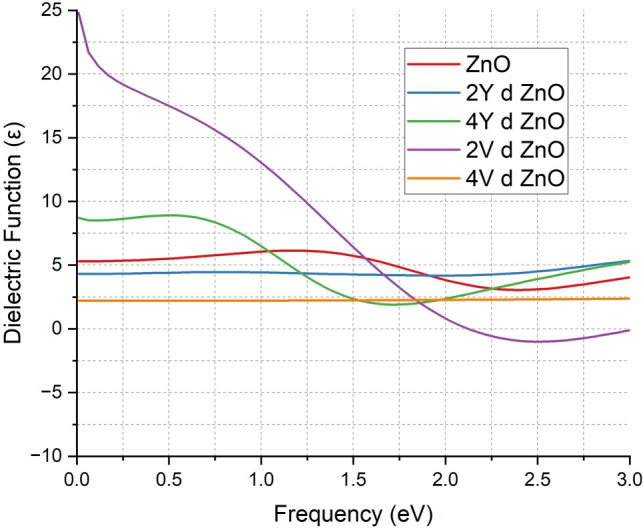
Comparison of dielectric function of ZnO & doped ZnO systems.

The dielectric function spectra provide important information on the polarization behavior and optical properties of ZnO to which Y and V have been doped as depicted in Fig. 10. The dielectric function of the undoped ZnO system (red curve) has a peak at 5 to 6 in the 0 to 3 eV frequency range, which is characteristic of semiconductors with moderate polarizability and weak interaction between free carriers.

The dielectric function is maintained at around 4 when ZnO is doped with 2Y atoms as shown by the blue curve. In 4Y doping, as indicated by the green curve, the dielectric function goes up and reaches its maximum at around 9. This tendency shows that an increase in the concentration of Y increases the responsiveness of the material to external electric fields, which is consistent with the observed increase in the conductivity and optical absorption.

The effect is even more pronounced in the V-doped systems. The 2V-doped ZnO (purple curve) has a maximum dielectric function at approximately 25, and the 4V-doped ZnO (orange curve) has a smaller value of approximately 2. Such high peaks suggest that the electronic polarizability of the material has changed significantly, which proves that the doped system and the incident electromagnetic fields are strongly interacting. Also, the negative values in the low frequency range indicate the occurrence of plasmonic behavior, which is characteristic of high-free electron concentration materials.

These results correspond to the available literature which suggests that the transition metal doping of ZnO leads to the improvement of the dielectric properties of the material because of the change in the electronic structure and the rise in the density of free carriers^18,19,48^. The general enhancement of dielectric functionality in all doped configurations ensures that the Y and V doping has a great effect on enhancing the optical and electronic nature of ZnO, which makes it a good candidate in terms of application in the field of optoelectronics, photonics and high-frequency dielectric applications.

ZnO doped with Y and V atoms was found to play a major role in improving the optical properties of the material i.e., its absorption, reflectivity, refractive index, conductivity, and dielectric function. As compared to the undoped system, the doped ZnO arrangements exhibit increased absorption at the visible spectrum, increased reflectivity, increased refractive index, increased electrical conductivity, and increased dielectric response. These enhancements validate that doping with Y and V effectively boosts the optoelectronic capabilities of ZnO, thereby enhancing its suitability for advanced optical and electronic applications.

### Thermal stability

**Fig. 11:**
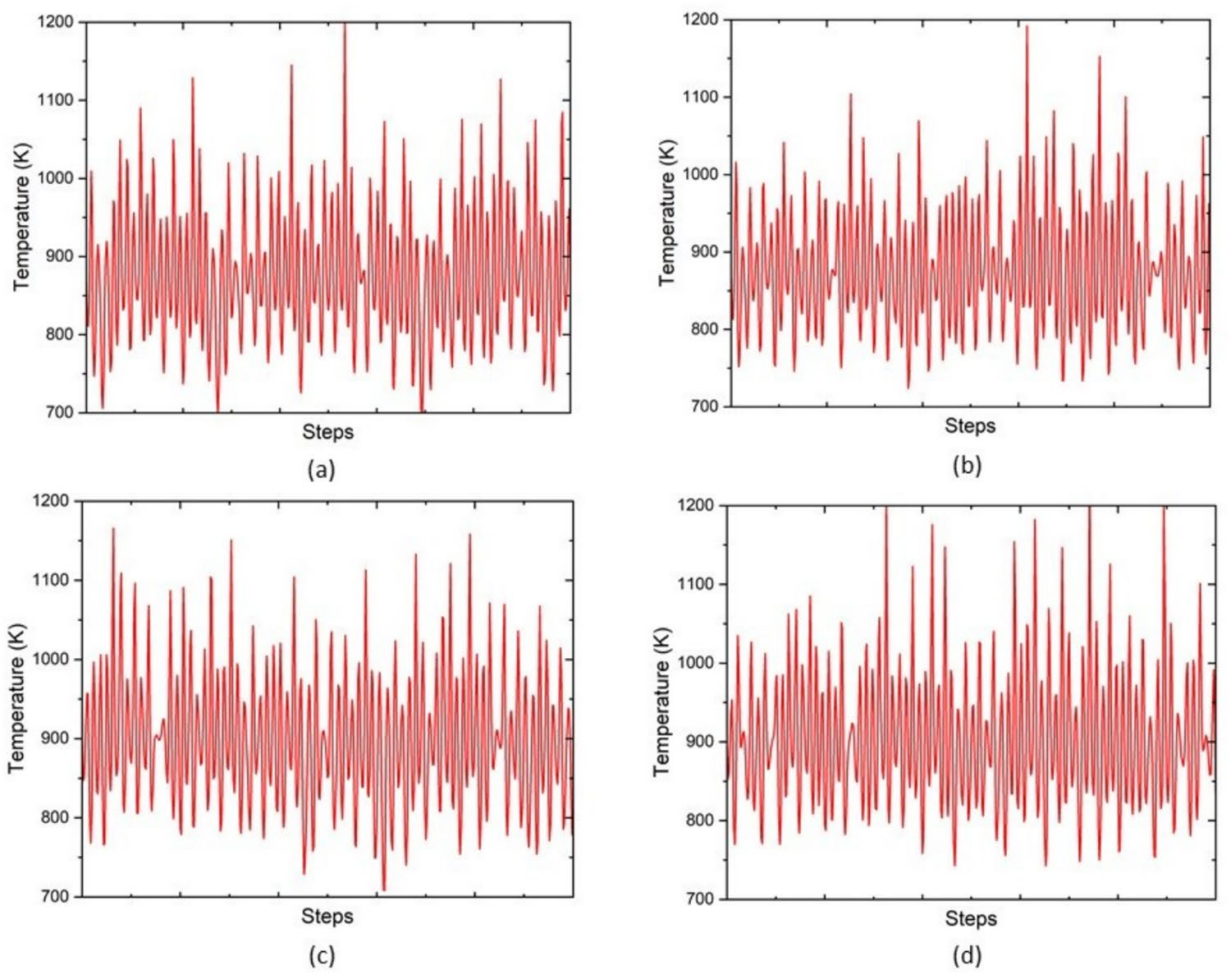
Comparison of thermal stability of ZnO & doped ZnO systems.

The configuration was determined using the NVT ensemble at 900 K through molecular dynamics with 5000 steps of 0.001 ps each. Figure 11a–d illustrates the temperature profile of the 2Y, 4Y, 2V and 4V configuration respectively. The 2Y and 4Y structures both have temperature differences within the 700–1200K range and within the 900K molecular dynamics simulations exhibit slight variation in temperature of the structure. This means that it is thermally stable even at high temperatures. On the same note, 2V and 4V systems exhibit identical temperature profiles when placed under identical conditions, and this suggests that V doping does not affect thermal integrity. All doped systems have their structural stability maintained at the high temperatures in the course of the simulation, which further contributes to their applicability in practice^18,19,48^.

## Conclusion

This paper provides a detailed computational study of the structural, electronic and optical properties of pure and doped ZnO systems, and the use of Y and V atoms as dopants at different concentration levels. The results show that doping has a significant effect on the properties of ZnO and each dopant influences the material in a specific manner. Structural analysis ensured that all the configurations, with the exception of 4V-doped system, were elastically stable. The 4V form exhibited mechanical instability owing to significant strains in the lattice which was caused by the large atomic radius of vanadium. On the other hand, the 2V-doped system did not lose its structural integrity but has a positive influence on the material properties. The analysis of electronic structure revealed that doping results in the decrease of the band gap and the rise of conductivity. The density of states also supported this observation and indicated that the density of electronic states near the Fermi level was high. The optical analysis of the systems such as absorption, reflectivity, refractive index, conductivity and dielectric function showed substantial improvements in the doped systems. The 2V-doped ZnO was found to have the most balanced and high performance in all optical measures of all configurations. It demonstrated good absorption in visible spectrum, great reflectivity, high refractive index, high optical conductivity and a great dielectric response, and maintained mechanical and structural stability. The XRD analysis confirmed that the cubic crystal structure of ZnO still exists in the 2V-doped system with slight changes in the position of the peaks indicating that the dopants have been successfully incorporated without affecting the crystallinity. Thermal stability tests were conducted at 900 K and discovered that the temperature changes were negligible, which means that the structure does not change under high temperatures. These findings also confirm the idea that doped ZnO systems are strong enough to be used in real-life optoelectronic applications.

In conclusion, while both Y and V doping improve the properties of ZnO, the 2V-doped configuration stands out as the most promising option. It presents an ideal blend of structural stability, improved electronic conductivity, and excellent optical performance, making it a strong candidate for applications in optoelectronics, transparent conductors, and photonic devices. Nevertheless, the limitations of this study could be the cell design and the basis set used. The approximation might possibly be improved with adjustments. It might be challenging to determine the exact doping percentage in the experimental setup, and it will be necessary to achieve the same level of precision and accuracy as in this computational study. The issues and challenges will be addressed in future work, along with the experimental realisation of the computational findings.

## Data Availability

Data will be provided by the corresponding author upon reasonable request.
